# Granulomatosis with polyangiitis in a patient treated with dabrafenib and trametinib for *BRAF* V600E positive lung adenocarcinoma

**DOI:** 10.1186/s12885-020-6661-6

**Published:** 2020-03-04

**Authors:** Anastasios Dimou, Gregory Barron, Daniel T. Merrick, Jason Kolfenbach, Robert C. Doebele

**Affiliations:** 1 Division of Medical Oncology, University of Colorado, Anschutz Medical Campus, 13001 E 17th Pl, Aurora, CO 80045 USA; 2 Division of Rheumatology, University of Colorado, Anschutz Medical Campus, 13001 E 17th Pl, Aurora, CO 80045 USA; 3 Department of Pathology, University of Colorado, School of Medicine, 13001 E 17th Pl, Aurora, CO 80045 USA; 4 Thoracic Oncology Research Initiative, Division of Medical Oncology, University of Colorado, School of Medicine, 12801 E. 17th Ave., MS 8117, Aurora, CO 80045 USA

**Keywords:** MAPK, Autoimmune side effects, MEK inhibitor, Pyrexia, P-ANCA

## Abstract

**Background:**

Dabrafenib and trametinib combination therapy is approved for the treatment of patients with *BRAF* V600E positive tumors including melanoma and lung cancer. The effect of BRAF and MEK inhibitors on the immune system is not fully understood although a number of case reports indicate autoimmune side effects related to the use of these drugs. Here, we discuss a case of a patient diagnosed with granulomatosis with polyangiitis (GPA) shortly after starting treatment with dabrafenib and trametinib for *BRAF* V600E positive metastatic lung adenocarcinoma.

**Case presentation:**

A 57 years old female patient was diagnosed with recurrent lung adenocarcinoma following initial lobectomy for early stage disease. A *BRAF* V600E mutation was identified at the time of recurrence and she received combination dabrafenib and trametinib therapy. Shortly after commencement of treatment, she developed persistent fevers necessitating withholding both drugs. Pyrexia continued and was followed by left vision loss and acute kidney injury. Further rheumatological workup led to the unifying diagnosis of GPA. The patient was then treated with rituximab for GPA to the present date while all antineoplastic drugs were held. Lung cancer oligoprogression was addressed with radiation therapy and has not required further systemic treatment whereas GPA has been controlled to-date with rituximab.

**Conclusions:**

This case report raises awareness among clinicians treating patients with lung cancer for the possibility of triggering a flare of autoimmune diseases like GPA in patients with *BRAF* V600E positive lung cancer receiving treatment with BRAF directed therapy.

## Background

*BRAF* V600E mutation causes aberrant MAPK signaling and drives 40–50% of melanomas [1, 2], 10% of colorectal cancers [3, 4],1–2% of lung adenocarcinomas [5, 6], 50% of the well differentiated thyroid carcinomas [7] and the vast majority of hairy cell leukemia cases [8] following the oncogene addiction disease model. Specific therapeutic targeting of BRAF V600E with mutation specific BRAF inhibitors in combination with MEK inhibitors is effective in melanomas with this molecular background [9]. Most recently, the combination of the BRAF V600E specific inhibitor dabrafenib and the MEK inhibitor trametinib was approved for the treatment of BRAF V600E positive lung cancer based on a phase II study showing PFS of 14.6 months and response rate of 64% [10].

Combination of dabrafenib with trametinib has an acceptable side effect profile with pyrexia reported as one of the most common grade 3 or higher toxicity, occurring in approximately 5–10% of the cases [10, 11]. Pyrexia is often accompanied by arthralgias and other musculoskeletal manifestations [12]. Dabrafenib monotherapy also carries this risk yet at a lower rate and presentation is typically less severe [10, 11]. Although the etiology of fever is poorly understood, it is well known that the thermostat is physiologically regulated by a cytokine surge including interleukin 1α and 1β (IL1α, IL1β), interleukin 6 (IL6) and tumor necrosis factor alpha (TNFα) [13]. These endogenous pyrogens were initially described as products of leucocytes, mostly monocytes, macrophages and neutrophils, in response to infectious stimuli [13, 14]. In addition, interferons, especially interferon alpha (IFNα) [14], interleukin 2 (IL2) [14], granulocyte macrophage colony stimulating factor (GM-CSF) [15] and the complement system [16] can induce fever either by direct hypothalamic effects or indirectly by inducing IL6 and TNFα.

The MAPK/ERK axis has important roles in multiple types of immune cells providing rationale for the pleiotropic effects of BRAF and MEK inhibitors on the innate and adaptive immune reactions [17]. The effect of MEK inhibition on the numbers and function of T cells has been controversial in the literature [18–21] with some reports indicating a complex, timing and context dependent relationship [21]. Interestingly, dabrafenib and trametinib combination treatment promotes the maturation of monocyte derived dendritic cells (moDCs) [22] which is also dependent on ERK signaling [23]. It is possible that the effect of ERK inhibition on immune cells drives febrile reactions in patients treated with dabrafenib and trametinib for BRAF V600E positive malignancies. Apart from pyrexia, an association of these drugs with diagnosis of a number of rheumatology conditions in several case reports [24–28] provides an intriguing link between ERK inhibition and autoimmunity.

Here, we present a case of a patient with *BRAF* V600E positive lung adenocarcinoma who was diagnosed with granulomatosis with polyangiitis (GPA) shortly after initiation of targeted therapy with dabrafenib and trametinib.

## Case presentation

The patient is a 57 years old never smoker female who initially received a clinical diagnosis of pneumonia. As symptoms failed to resolve with antimicrobials, a subsequent CT scan of the chest revealed a partially cavitary mass in the right lower lung lobe. This imaging finding was followed with CT scans for two years at an outside facility showing slow growth. Eventually, a CT guided biopsy revealed mucinous adenocarcinoma of the lung with predominant lepidic pattern. A PET CT and MRI of the brain at the time did not show any other disease sites and she received a right lower lobectomy which confirmed the diagnosis and the stage as pT2bpN0M0 (IIA). Following surgery, the patient received adjuvant chemotherapy with carboplatin and paclitaxel for four cycles.

She carried a diagnosis of idiopathic autoimmune hearing loss, that had been successfully treated with mycophenolate mofetil. Her family history included lung cancer in both of her parents and her sister, all smoking related, as well as breast cancer in her maternal aunt.

**Fig. 1:**
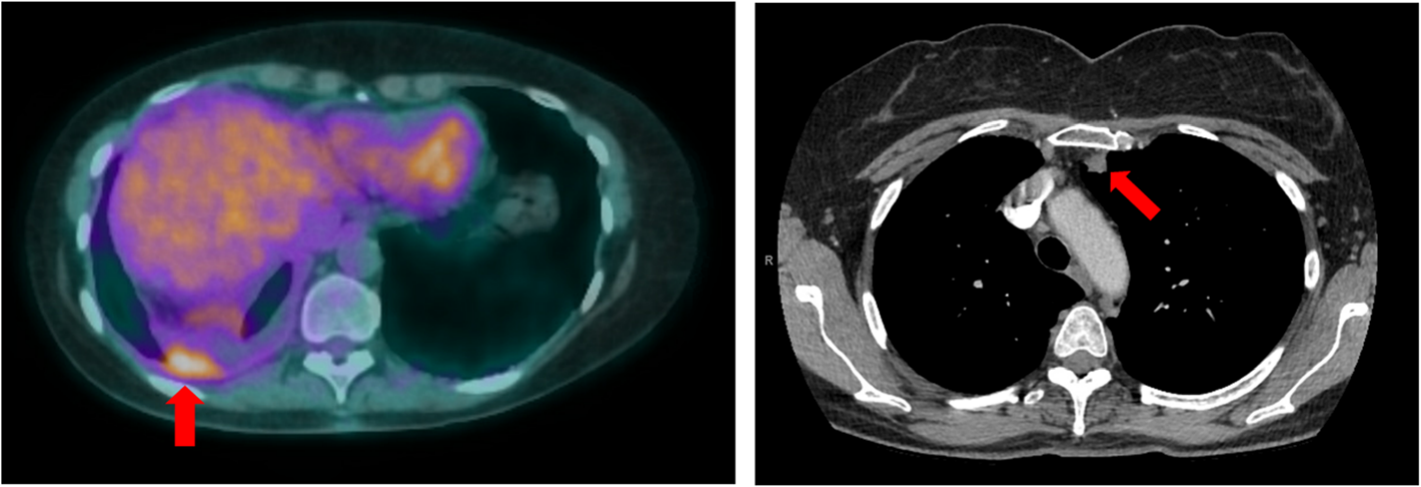
Sites of disease on recurrence. Pleural thickening with metabolic activity on the right was biopsied and pathology review confirmed mucinous adenocarcinoma with predominant lepidic pattern. In 3 months following radiation therapy of the recurring pleural lesion, there were new lung nodules identified on CT scan. One of these nodules is shown at the left upper lobe on the right

A year after her surgery, disease recurrence was documented on imaging in the right pleura. The same neoplasm was identified upon pathology review of a right pleural biopsy and she received local radiation therapy as salvage treatment. Follow up imaging in 3 months identified new lung nodules and the patient was referred to our institution. Figure 1 shows the metabolically avid right pleural thickening that was radiated and one of the lung nodules at the time of disease recurrence following radiation. Molecular analysis of the original lobectomy material with next generation sequencing revealed a *BRAF* V600E mutation. Subsequently, she was initiated on combination of dabrafenib and trametinib treatment in the context of a clinical trial.

While on the experimental drugs for two weeks, she experienced significant fatigue, persistent fevers up to 38 °C and generalized myalgias necessitating holding dabrafenib and trametinib. Nevertheless, symptoms persisted and infectious and rheumatology workups were initiated at the time. In addition, three weeks after stopping dabrafenib and trametinib, she was admitted for left eye vision loss and acute kidney injury. An ophthalmology exam with eye dilation indicated left central artery occlusion. Additional data from her history, exam and laboratory evaluation revealed the following: a history of recurrent sinusitis, acute onset visual loss and renal insufficiency during the current admission, and evidence of a saddle-nose deformity on exam which the patient believed was present for several years prior. She subsequently received a unifying diagnosis of granulomatosis with polyangiitis (GPA) on the basis of these findings as well as high-titer characteristic antibodies (p-ANCA titer 1:640, myeloperoxidase antibody > 30). Other lab results including rheumatology workup are shown in Table 1. Due to acute vision loss, giant cell arteritis was considered and a temporal artery biopsy was obtained and found negative. Her acute vision loss and creatinine elevation were thought secondary to retinal and renal vascular involvement by GPA. Nevertheless, review of the pleural biopsy and the resection specimen by pathology in retrospect, did not reveal any granulomatous change or vasculitis. She was initiated on rituximab, corticosteroids were successfully tapered, and further anti-neoplastic drugs were held. Lung cancer was followed clinically with scans.

**Table 1:** Laboratory Data

Variable (normal range)	1.5 months prior to admission	At the time of hospital admission	2.5 months following admission
Hematocrit (%) (35.7–46.7)	41	30.1	38.3
Hemoglobin (g/dl) (12.1–16.3)	13.2	9.8	12.3
White Cell Count (*10^9^/L) (4–11.1)	6.9	7.5	8.4
Differential (%)			
Neutrophils	74.9	80.5	85.6
Band forms	0	0.6	0.6
Lymphocytes	13.7	6.7	7.0
Monocytes	9.1	9.9	5.6
Mean Corpuscular volume (fL) (80–100)	91.1	87.5	97
Erythrocyte count (*10^12^/L) (4.18–5.64)	4.5	3.44	3.95
Potassium (mmol/L) (3.5–5.1)	4.4	4.1	4.5
Sodium (mmol/L) (133–145)	137	136	135
Chloride (mmol/L) (98–108)	103	107	103
Blood Urea Nitrogen (mg/dl) (7–25)	16	22	32
Creatinine (mg/dl) (0.6–1.2)	0.98	1.47	1.86
Calcium (mg/dl) (8.6–10.3)	9.2	8.6	9.2
Glucose (mg/dl) (70–199)	99	162	124
Alkaline Phosphatase (U/L) (39–117)	114	96	64
Alanine Aminotransferase (U/L) (7–52)	12	13	19
Aspartate Aminotransferase (U/L) (12–39)	30	17	19
Bilirubin total (mg/dl) (0.1–1.3)	0.7	0.6	0.7
Total protein (g/dl) (6.4–8.9)	7.1	6.6	6.4
Albumin (g/dl) (3.5–5.7)	3.6	2.9	3.8
ESR (mm/hr) (0–20)		75	12
Anti DNA Ab		Negative	
Anti Centromere Ab		Negative	
Anti RNP Ab		Negative	
Anti Smith Ab		Negative	
Anti SSA Ab		Negative	
Anti SSB Ab		Negative	
ANA		> 1:320, speckled	
Anti MPO Ab (0–20 units)		> 30	> 30
C-ANCA		Negative	Negative
P-ANCA		1:640	1:80
C3 omplement (mg/dl) (87–200)		110	
C4 Complement (mg/dl) (19–52)		25.1	

A year after diagnosis of GPA, a growing lung nodule was proven with biopsy to be malignant and was treated with SBRT. To-date, three years following GPA diagnosis and lung cancer recurrence, both conditions remain controlled without any further systemic therapy for lung cancer and while she continues on rituximab for GPA.

## Discussion and conclusions

A number of case reports have described a potential association between autoimmune disease and inhibition of ERK by dabrafenib and trametinib. These drugs are increasingly used for the treatment of several malignancies harboring *BRAF* V600E mutations, and autoimmune manifestations that have been reported with their use include pneumonitis [29], dermatomyositis [26] and panniculitis [24, 25]. GPA is a necrotizing vasculitis affecting small vessels with concomitant granuloma formation and inflammation mainly of the respiratory system. It belongs to the group of ANCA associated vasculitides (AAV) that also includes microscopic polyangiitis, eosinophilic granulomatosis with polyangiitis and drug induced AAV [30]. While characteristic histopathology can be identified on tissue biopsy, diagnostic yield can vary according to site (15–25% from biopsies of the nasal and sinus passages) and surgical technique (lower sensitivity for transbronchial biopsy compared to open lung biopsy) [31, 32]. A positive ANCA antibody (cANCA or pANCA) in the setting of concurrent antigen-specific antibody presence (PR-3 antibody or myeloperoxidase antibody, respectively) confers greater than 90% specificity for the diagnosis.

In the case presented herein, it is likely the patient was experiencing symptoms of GPA (recurrent sinusitis, hearing loss responsive to immunosuppression) years prior to formal diagnosis, based upon the presence of a chronic saddle nose deformity. None-the-less, shortly after institution of dabrafenib and trametinib, she suffered an acute exacerbation of end-organ disease at the eyes and kidneys, prompting a thorough evaluation that led to a definitive diagnosis of GPA. In conclusion, this is the first case report to link a flare of GPA to MAPK pathway inhibition. Current understanding of the multifaceted and context dependent link between ERK pathway and autoimmunity is incomplete [33, 34]. While the connection might be coincidental, the temporal association and the known role of ERK in the immune system, as well as the association of these medications with other autoimmune manifestations in the literature, make a drug effect plausible. Alternatively, GPA might be a paraneoplastic manifestation of lung adenocarcinoma and response to dabrafenib and trametinib might have released tumor-associated antigens that led to GPA flare. This latter possibility is also supported by the co-existence of autoimmune related symptoms and the original lung mass for years before the formal diagnosis of GPA. Given that GPA was thought to be present prior to treatment with dabrafenib and trametinib, a triggering rather than a causative effect could have taken place. This case, along with others in the literature, suggests that inhibition of ERK signaling may be associated with the development of autoimmune clinical phenotypes, and further research in this area is needed.

## Abbreviations

BRAF: V-Raf Murine Sarcoma Viral Oncogene Homolog B1
CT: Computerized tomography
ERK: Extracellular signal regulated kinase
GM-CSF: Granulocyte-macrophage colony stimulating factor
GPA: Granulomatosis with polyangiitis
IFNα: Interferon alpha,
IL1α: Interleukin 1 alpha
IL1β: Interleukin 1 beta
IL2: Interleukin 2
IL6: Interleukin 6
MAPK: Mitogen-activated protein kinase
MEK: Mitogen-Activated Protein Kinase Kinase
MoDCs: Monocyte derived dendritic cells
MRI: Magnetic resonance imaging
p-ANCA: Perinuclear antineutrophil cytoplasmic antibodies
PET: Positron emission tomography
PFS: Progression Free Survival
SBRT: Stereotactic body radiation therapy
TNFα: Tumor Necrosis Factor alpha
